# Enhancing pathogen description and antibiotic regimen selection in community-acquired pneumonia through RT-qPCR assays

**DOI:** 10.3389/fmicb.2024.1409065

**Published:** 2024-06-11

**Authors:** Na Zhao, Hongyu Ren, Yingmiao Zhang, Yan Jiang, Jianping Deng, Luxi Jiang, Zhongxin Lu, Tian Qin

**Affiliations:** ^1^National Key Laboratory of Intelligent Tracking and Forecasting for Infectious Diseases, National Institute for Communicable Disease Control and Prevention, Chinese Center for Disease Control and Prevention, Beijing, China; ^2^The Central Hospital of Wuhan, Tongji Medical College, Huazhong University of Science and Technology, Wuhan, China; ^3^Center for Disease Control and Prevention, Zigong, China; ^4^Department of Respiratory Medicine, Zhejiang Provincial People’s Hospital, People’s Hospital of Hangzhou Medical College, Hangzhou, China

**Keywords:** community-acquired pneumonia, respiratory pathogens, RT-qPCR assay, antibiotic therapy, severe CAP

## Abstract

**Background:**

Adults with community-acquired pneumonia (CAP) in China suffer high morbidity. CAP is caused by a multitude of pathogens; however, pathogen-directed clinical symptoms are often lacking. Therefore, patients lacking an accurate microbiological diagnosis are administered with empirical antimicrobials.

**Methods:**

We collected bronchoalveolar lavage fluid, as well as clinical and laboratory data from 650 adult patients with CAP admitted to three hospitals in Hubei, Sichuan, and Zhejiang provinces in China. Specimens were cultured and tested using real-time reverse transcription qPCR (RT-qPCR) assays for the presence of 42 respiratory bacteria and viruses. CAP was investigated with respect to regions, genders, and age and patterns of infections or co-infections. Employing clinical guidelines adapted for diagnosis, we assessed retrospectively the appropriate pathogen-directed therapy and compared it with the initial empirical therapies.

**Results:**

Our study identified that 21.38% (139/650) of the patients were classified as having Severe CAP (S-CAP), with a higher prevalence among males, older adults, and during the warm season. Bacterial pathogens were detected in 35.53% (231/650) of cases. *K. pneumoniae*, *H. influenzae*, and *S. aureus* were the most prevalent bacteria across different demographics and regions. Viral pathogens were found in 48.76% (317/650) of patients Epstein-Barr, Human rhinovirus, and Cytomegalovirus were the most common viruses. Co-infections were present in 24.31% (158/650) of cases, with viral-bacterial co-infections being the most frequent. The RT-qPCR demonstrated significantly higher detection rates for key pathogens compared to standard culture methods. It showed potential in optimizing antimicrobial prescriptions by allowing for de-escalation in 18.30% (95/518) of patients, among which reducing the number of excessive antibiotics mainly comprised decreasing the use of 2nd or 3rd generation cephalosporins (5.79%, 30/518) and β-lactamase inhibitor combinations.

**Conclusion:**

The study highlights the significant burden of S-CAP, particularly among specific demographics and seasons. The prevalence of bacterial and viral pathogens, along with the high rate of co-infections, emphasizes the need for comprehensive diagnostic approaches. The RT-qPCR assays emerge as a superior diagnostic tool, offering enhanced pathogen detection capabilities and facilitating more precise antimicrobial therapy. This could lead to improved patient outcomes and contribute to the rational use of antimicrobials, addressing the growing concern of antibiotic resistance.

## 1 Introduction

The incidence of CAP (an infectious condition characterized by inflammation of the lung parenchyma that occurs outside of hospital settings) varies geographically; in European and North American countries, it ranges from 5 to 11 cases per 1,000 person-years, while in the USA, it is reported at 2.5 cases per 1,000 person-years (Lim et al., 2009; Welte and Köhnlein, 2009; Sun et al., 2020). Notably, the incidence in China was recorded as 7.13 cases per 1,000 person-years in 2016, highlighting a relatively high rate of occurrence (Jain et al., 2015).

CAP is caused by a wide range of pathogens; therefore, patients with moderate or S-CAP clinical symptoms are treated using broad-spectrum antimicrobials before a definitive diagnosis, which should be de-escalated to pathogen-specific agents post-diagnosis (Tao et al., 2012; McEwen and Collignon, 2018). Unfortunately, in clinical practice, de-escalation of antibiotics often does not occur. The reason is that clinical microbiology laboratories rely heavily on bronchoalveolar lavage fluid or sputum culture methods, which are time-consuming, less sensitive, and require rigorous culture conditions, prohibiting a timely diagnosis. Using only the results of culture, a pathogen might only be detected in 30–40% of patients with CAP (Musher et al., 2013; Musher and Thorner, 2014). Additionally, CAP patients often experience co-infections, which increase the complexity of treatment. Therefore, patients lacking an accurate microbiological diagnosis receive empirical antimicrobials (Zhao et al., 2015; Duong et al., 2018). Research has revealed the requirement for more sensitive and timelier methods for the microbiological diagnosis of pathogens in CAP (Caliendo, 2011; Zhang et al., 2015, 2018; Sun et al., 2021).

Thus, we need new techniques to rapidly and accurately identify CAP causative agents. Molecular testing has certain advantages over traditional methods (cultures, serology for atypical bacteria, direct immunofluorescence tests, and rapid antigen tests), such as increased speed, higher sensitivity, and multiple pathogen detection, which markedly augment CAP microbial identification efficiency. Moreover, for atypical pathogens that need rigorous culture conditions and respiratory viruses, identification via molecular methods is crucial. The application of the RT-qPCR assays to respiratory specimens allows rapid screening for a plethora of pathogens in the minimum number of reactions. This has led to the RT-qPCR assay method becoming increasingly important in microbial detection (Templeton et al., 2003; Gadsby et al., 2016).

Through laboratory testing of bronchoalveolar lavage fluid from 650 adult CAP patients admitted to three hospitals in China, we aimed to enhance the detection rate of pathogens, explore the causal and epidemiological characteristics by region, gender, and age, to better delineate the distribution pattern of CAP in the community, and its association with severe diseases. Consequently, this will guide early antimicrobial treatment.

## 2 Materials and methods

### 2.1 Subjects

Between July 2020 and September 2021, a total of 650 bronchoalveolar lavage fluid specimens from 650 adult patients with CAP were included in this study. All patients were older than 18 years. The patients were from Hubei (372), Sichuan (97), and Zhejiang (181) hospitals. Patients diagnosed with CAP were included if they exhibited signs of acute respiratory tract infections (such as fever or chills, abnormal white blood cell counts, newly developed cough or sputum production, chest pain, difficulty breathing, rapid breathing, and abnormal lung examination) along with findings consistent with pneumonia on chest radiography, and received a pneumonia diagnosis within 24 h of hospital admission (Cao et al., 2018). Clinical data (patient age, sex, length of hospital stay, results of lavage fluid culture, and empirical antibiotics administered) were collected retrospectively from the patients’ hospital records.

### 2.2 Classification of mild versus severe cases

We divided the CAP cases into two groups: mild and severe CAP. All the cases were classified as Middle CAP (M-CAP) unless the patients met any of the major criteria or three or more minor criteria, in which case they were diagnosed as having S-CAP. Major criteria: (1) Requiring tracheal intubation and mechanical ventilation; (2) Septic shock, and still in need of vasoactive drugs after active fluid resuscitation. Minor criteria: (1) Respiratory rate (RR) ≥ 30 bpm; (2) Oxygenation index ≤250 mm Hg (1 mm Hg = m Hg kPa); (3) Infiltrates in multiple lung lobes; (4) Disturbance of consciousness and (or) disorientation; (5) Blood urea nitrogen (BUN) ≥; (5 mmol/L; (6) Systolic blood pressure (SBP) < mo mm Hg, requiring active fluid resuscitation (Cao et al., 2018).

### 2.3 Microbiological culture

Common respiratory bacteria were detected using standard biochemical and microbiological methods from lavage fluid culture (Sterback et al., 2013). Clinical specimens were inoculated onto Chocolate agar (Oxoid, Basingstoke, UK), blood agar (Oxoid), and MacConkey agar (Beijing Land Bridge, Beijing, China), and incubated for 24 h in a 5% CO_2_ atmosphere at 37 °C. Single colonies were selected for purification and culture. Microbial isolates were identified using Mass spectrometry (M-DISCOVER 100, Zhuhai Meihua, Zhuhai, China).

### 2.4 RT-qPCR assays

A RNeasy Mini Kit (Qiagen, Hilden, Germany) was used to extract total RNA from an aliquot (0.2 mL) of the lavage fluid. The TaqMan Array Card for comprehensive respiratory tract microbiota analysis (Thermo Fisher Scientific) was run on the QuantStudio 7 Flex platform (Thermo Fisher Scientific, Waltham, MA, USA), following the manufacturer’s instructions. Briefly, 25 μL of RNA was added to 25 μL of TaqMan Fast Virus 1-step mastermix (Thermo Fisher Scientific) and 50 μL of RNase-free water, mixed, and loaded onto the TaqMan array card. Additionally, one PBS sample was processed as the negative control using the same reagents and equipment in the same laboratory. These chips dispense 48 PCR mixes and 8 samples into individual wells, resulting in 384 individual reactions. Quantitative RT-qPCR was carried out as follows: 50°C for 15 min (reverse transcription step); 95°C for 10 min; and then 95°C for 3 s followed by 60°C for 1 min (40 cycles), with a fluorescence reading taken on the FAM channel at each cycle. A positive result comprised detection of a distinct exponential amplification curve whose cycle threshold (CT) value was less than or equal to 35. The RT-qPCR was applied for 42 target pathogens (including 12 bacteria, 1 fungus, and 29 viruses) as shown in Supplementary Table 1. The card also included four controls: Xeno (synthetic RNA control, used for nucleic acid isolation and exogenous process control for RNA recovery, reverse transcription, pre-amplification and PCR), RNase P, 18S, and *Bacillus atrophaeus.*

### 2.5 Estimating the impact of RT-qPCR assay on the antimicrobial prescription

Based on the molecular identification results (Supplementary Table 2), for each patient, the administered empirical treatment was compared with the anti-infective therapy for CAP that should have been administered (Cao et al., 2018).

### 2.6 Statistical analyses

Pearson’s χ2 test was used to evaluate the differences in infection of pathogens among patients with CAP. SPSS software version 25.0 was used to perform all the statistical analyses and differences with a *P* < 0.05 were considered statistically significant.

## 3 Results

### 3.1 General characteristics of the patients

Among all patients, 21.38% (139/650) of cases were defined as S-CAP, while 78.61% (511/650) were classified as M-CAP. The proportion of S-CAP was higher in males (67.63% [94/139] vs. 51.86% [265/511]), in older adults age ≥ 65; (64.03% [89/139] vs. 38.94% [199/511]), and during the warm season (74.82% [104/139] vs. 44.42% [227/511], all *p* < 0.001) compared to M-CAP (Table 1).

**TABLE 1:** Characteristics of the recruited patients with CAP (*n* = 650).

Characteristics	Total (*N* = 650)	M-CAP (*N* = 511)	S-CAP (*N* = 139)	*p*-value*
Region				<0.001
HuBei	372 (57.23%)	300 (58.71%)	72 (51.80%)	
Zhejiang	181 (27.85%)	180 (35.23%)	1 (0.72%)	
Sichuan	97 (14.92%)	31 (6.07%)	66 (47.48%)	
Sex				<0.001
Male	359 (55.13%)	265 (51.86%)	94 (67.63%)	
Female	291 (44.87%)	246 (48.14%)	45 (32.37%)	
Age(yr)				<0.001
Age < 65	362 (55.69%)	312(61.06%)	50 (35.97%)	
65 ≤ age	288 (44.31%)	199 (38.94%)	89 (64.03%)	
Season of infection†				<0.001
Warm	331 (50.92%)	227 (44.42%)	104 (74.82%)	
Cold	319 (49.08%)	284 (55.58%)	35 (25.18%)	

*p-values were compared between the M-CAP group and S-CAP group.

^†^The season of infection corresponds to the 6 months with the highest average temperature each year in the city where each CAP patient is located, and the rest considered the cold season.

### 3.2 Pathogens

In the examination of bacterial screening among 650 patients, it was found that 231 cases (35.53%) had at least one positive bacterial detection. The highest rate was determined in the Hubei region ([171/372] 45.97%), followed by 31.96% (31/97) in the Sichuan region and 16.02% (29/181) in the Zhejiang region (Table 2). Significantly higher bacterial positive rates were seen in the Warm season compared to the Cold season (41.39% [137/331] vs 29.46% [94/319], *p* < 0.05). There may be some variations in the ranking of the top three bacterial pathogens across different age groups, genders, seasons, and regions (Hubei and Zhejiang). However, overall, *K. pneumoniae*, *H. influenzae*, and *S. aureus* remained the most prevalent bacterial pathogens. In the Sichuan region, the top three pathogens were *H. influenzae*, *S. pneumoniae*, and *S. aureus*.

**TABLE 2:** Positive rate of respiratory pathogens among patients with CAP (*n* = 650).

	Region				Sex			Age			Season of infection			Total
	Hubei	Zhejiang	Sichuan	*P-*value	Male	Female	*P*-value	Age < 65	65 ≤ age	*P-*value	Warm	Cold	*P-*value	
Bacteria	171/372 (45.97%)	29/181 (16.02%)	31/97 (31.96%)	0.001	135/359 (37.60%)	96/291 (32.99%)	0.222	125/362 (34.53%)	106/288 (36.81%)	0.565	137/331 (41.39%)	94/319 (29.46%)	0.013	231/650 (35.53%)
*K. pneumoniae*	77 (20.70%)	7 (3.87%)	6 (6.19%)	0.001	64 (17.83%)	26 (8.93%)	0.247	46 (12.71%)	44 (15.28%)	0.353	54 (16.31%)	36 (11.29%)	0.139	90 (13.85%)
*H. influenzae*	48 (12.90%)	19 (10.50%)	18 (18.56%)	0.119	42 (11.70%)	43 (14.78%)	0.305	50 (13.81%)	35 (12.15%)	0.524	49 (14.80%)	36 (11.29%)	0.334	85 (13.08%)
*S. aureus*	46 (12.37%)	19 (10.50%)	9 (9.28%)	0.558	45 (12.53%)	29 (9.97%)	0.499	41 (11.33%)	33 (11.46%)	0.968	42 (12.69%)	32 (10.03%)	0.465	74 (11.38%)
*M. catarrhalis*	35 (9.41%)	1 (0.55%)	2 (2.06%)	0.001	23 (6.41%)	15 (5.15%)	0.049	19 (5.25%)	19 (6.60%)	0.472	23 (6.95%)	15 (4.70%)	0.324	38 (5.85%)
*S. pneumoniae*	12 (3.23%)	4 (2.21%)	9 (9.28%)	0.007	9 (2.51%)	16 (5.50%)	0.328	18 (4.97%)	7 (2.43%)	0.093	17 (5.14%)	8 (2.51%)	0.12	25 (3.85%)
*M. Pneumonia*	4 (1.08%)	0	2 (2.06%)	0.154	5 (1.39%)	1 (0.34%)	1	3 (0.83%)	3 (1.04%)	1.000	5 (1.51%)	1 (0.31%)	0.273	6 (0.92%)
*L.pneumophila*	9 (2.42%)	0	0	0.026	5 (1.39%)	4 (1.37%)	1	7 (1.93%)	2 (0.69%)	0.313	4 (1.21%)	5 (1.57%)	0.867	9 (1.38%)
Bordetella	2 (0.54%)	1 (0.55%)	2 (2.06%)	0.281	3 (0.84%)	2 (0.69%)	0.028	3 (0.83%)	2 (0.69%)	1.000	3 (0.91%)	2 (0.63%)	1	5 (0.77%)
Virus	218/372 (58.60%)	57/181 (31.49%)	42/97 (43.30%)	0.001	189/359 (52.65%)	128/291 (43.99%)	0.028	157/362 (43.37%)	160/288 (55.56%)	0.002	155/331 (46.83%)	162/319 (50.78%)	0.054	317/650 (48.76%)
Epstein-Barr (EB)	100 (26.88%)	31 (17.13%)	26 (26.80%)	0.014	93 (25.91%)	64 (21.99%)	0.621	65 (17.96%)	92 (31.94%)	0.000	87 (26.28%)	70 (21.94%)	0.446	157 (24.15%)
Human rhinovirus (HRV)	76 (20.43%)	12 (6.63%)	4 (4.12%)	0.058	53 (14.76%)	39 (13.40%)	0.049	52 (14.36%)	40 (13.89%)	0.852	52 (15.71%)	40 (12.54%)	0.436	92 (14.15%)
Cytomegalovirus (CMV)	50 (13.44%)	15 (8.29%)	11 (11.34%)	0.144	50 (13.93%)	26 (8.93%)	0.662	26 (7.18%)	50 (17.36%)	0.000	34 (10.27%)	42 (13.17%)	0.136	76 (11.69%)
Mumps (MuV)	45 (12.10%)	15 (8.29%)	4 (4.12%)	0.036	37 (10.31%)	27 (9.28%)	0.088	37 (10.22%)	27 (9.38%)	0.711	34 (10.27%)	30 (9.40%)	0.952	64 (9.85%)
Respiratory syncytial virus B (RSVB)	16 (4.30%)	2 (1.10%)	0	0.016	8 (2.23%)	10 (3.44%)	0.988	11 (3.04%)	7 (2.43%)	0.635	10 (3.02%)	8 (2.51%)	0.81	18 (2.77%)
Human metapneumovirus (HMPV)	7 (1.88%)	3 (1.66%)	0	0.4	5 (1.39%)	5 (1.72%)	0.721	6 (1.66%)	4 (1.39%)	1.000	3 (0.91%)	7 (2.19%)	0.257	10 (1.54%)
Human herpesvirus 6 (HHV-6)	9 (2.42%)	0	0	0.029	6 (1.67%)	3 (1.03%)	0.855	6 (1.66%)	3 (1.04%)	0.739	7 (2.11%)	2 (0.63%)	0.239	9 (1.38%)
Adenovirus (ADV)	3 (0.81%)	0	0	0.724	1 (0.28%)	2 (0.69%)	1	3 (0.83%)	0	0.333	1 (0.30%)	2 (0.63%)	0.923	3 (0.46%)
Bochavirus (HBOV)	0	0	1 (1.03%)	0.147	1 (0.28%)	0	1	1 (0.28%)	0	1.000	1 (0.30%)	0	1	1 (0.15%)
Influenza B virus (IBV)	1 (0.27%)	0	0	1	1 (0.28%)	0	0.691	1 (0.28%)	0	1.000	1 (0.30%)	0	1	1 (0.15%)
Parainfluenza (PIV)	12 (3.23%)	0	2 (2.06%)	0.042	7 (1.95%)	7 (2.41%)	1	5 (1.39%)	9 (3.13%)	0.130	10 (3.02%)	4 (1.25%)	0.157	14 (2.15%)
PIV-1	3 (0.81%)	0	0	0.724	2 (0.56%)	1 (0.34%)	1	0	3 (1.04%)	0.173	2 (0.60%)	1 (0.31%)	1	3 (0.46%)
PIV-3	6 (1.61%)	0	2 (2.06%)	0.104	4 (1.11%)	4 (1.37%)	0.855	4 (1.10%)	4 (1.39%)	1.000	5 (1.51%)	3 (0.98%)	0.843	8 (1.23%)
PIV-4	3 (0.81%)	0	0	0.724	1 (0.28%)	2 (0.69%)	1	1 (0.28%)	2 (0.69%)	0.844	3 (0.91%)	0	0.288	3 (0.46%)
Human coronavirus (HCoV)	6 (1.61%)	4 (2.21%)	0	0.496	5 (1.39%)	4 (1.37%)	1	2 (0.55%)	7 (2.43%)	0.090	4 (1.21%)	6 (1.88%)	0.62	10 (1.54%)
HCoV-229E	2 (0.54%)	1 (0.55%)	0	1	2 (0.56%)	1 (0.34%)	0.855	0	3 (1.04%)	0.173	2 (0.60%)	1 (0.31%)	1	3 (0.46%)
HCoV-HKU1	2 (0.54%)	1 (0.55%)	0	1	1 (0.28%)	2 (0.69%)	1	1 (0.28%)	2 (0.69%)	0.844	0	3 (0.94%)	0.209	3 (0.46%)
HCoV-OC43	2 (0.54%)	1 (0.55%)	0	1	1 (0.28%)	1 (0.34%)	1	0	2 (0.69%)	0.197	1 (0.30%)	2 (0.63%)	0.923	3 (0.46%)
HCoV-NL63	0	1 (0.55%)	0	1	1 (0.28%)	0	0.001	1 (0.28%)	0	1.000	1 (0.30%)	0	0.013	1 (0.15%)

Among the 650 patients with CAP tested for all virus pathogens, 317 cases (48.76%) had at least one positive detection. The highest rate was determined in the Hubei region (218/372, 58.60%), followed by 43.30% (42/97) in the Sichuan region and 31.49% (57/181) in the Zhejiang region. It is worth noting that the positive rate was significantly higher in patients aged 65 and above compared to younger patients (55.56% [160/288] vs 43.37% [157/362]), in males than females (52.65% [189/359] vs 43.99% [128/291]); all *p* < 0.05). Variations in the ranking of the top three viral pathogens were observed across different groups, in males and elderly patients, as well as in regions (Hubei and Sichuan). Nevertheless, EB, HRV and CMV remained the most common viral pathogens overall. In the Zhejiang region, among female patients and younger patients, the top three were EB, CMV, and MuV (Table 2).

**FIGURE 1:**
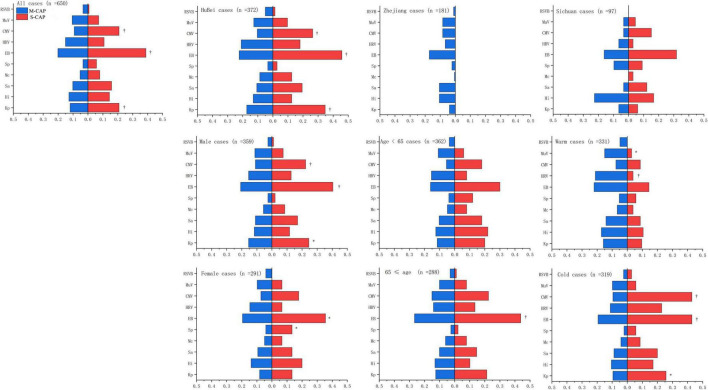
Comparison of the positivity rates for major pathogens between S-CAP and M-CAP. Kp, *K. pneumoniae*; HI, *H. influenzae*; Sa, *S. aureus*; Mc, *M. catarrhalis*; Sp, *S. pneumoniae;* EB, Epstein-Barr virus; HRV, Human Rhinovirus; CMV, Cytomegalovirus; MuV, Mumps; RSVB, Respiratory syncytial virus B.

Among the 511 M-CAP patients, 175 (34.24%) tested positive for at least one bacterial pathogen, while 236 (46.18%) tested positive for at least one viral pathogen. In contrast, among the 139 S-CAP patients, 81 (58.27%) tested positive for at least one bacterial pathogen, and 56 (40.28%) tested positive for at least one viral pathogen. Notably, in S-CAP patients, the positivity rates for EB (38.85% [54/139] vs 20.16% [103/511]), CMV (20.86% [29/139] vs 9.20% [47/511]), and *K. pneumoniae* (20.86% [29/139] vs 11.94% [61/511]) were significantly higher compared to M-CAP. The same trend was observed during the Cold season, among males, and in the Hubei region. Among female patients, S-CAP showed a significantly higher positivity rate for *S. pneumoniae* (13.33% [6/45] vs 4.06% [10/246]) compared to M-CAP, which was not observed in other groups. Noteworthy is the observation that during the Warm season, M-CAP patients exhibited significantly higher positivity rates for HRV (21.15% [48/227] vs 3.85% [4/104]) and MuV (14.98% [34/227] vs 2.88% [3/104]) compared to S-CAP (Figure 1).

### 3.3 Comparison of the RT-qPCR assays with culture for pathogen detection

Based on the data from Table 3, the RT-qPCR method demonstrated significantly higher detection rates for specific pathogens compared to standard culture-based methods, all with *p* < 0.001. Notably, the diagnostic yields for *K. pneumoniae*, *H. influenzae*, *S. aureus, S. pneumoniae*, and *M. catarrhalis* were 89.80, 97.14, 96.15, 100.00, and 100.00%. Importantly, RT-qPCR could detect viruses, whereas the culture-based methods could not.

**TABLE 3:** Comparison of the RT-qPCR and culture based methods for bacterial detection.

Organism	RT-qPCR	Culture		RT-qPCR diagnostic yield^†^	*p*-value
		Positive	Negative		
		*N* (%)	*N* (%)		
*K. pneumoniae*	Positive	18 (2.77%)	70 (10.77%)	89.80%	<0.0001
	Negative	10 (1.54%)	552 (84.92%)		
*S. aureus*	Positive	15 (2.31%)	53 (8.15%)	97.14%	<0.0001
	Negative	2 (0.31%)	580 (89.23%)		
*S. pneumoniae*	Positive	4 (0.62%)	21 (3.23%)	96.15%	<0.001
	Negative	1 (0.15%)	624 (96.00%)		
*H. influenzae*	Positive	1 (0.15%)	83 (12.77%)	100.00%	<0.0001
	Negative	0	566 (87.08%)		
*M. catarrhalis*	Positive	0	38 (5.85%)	100.00%	<0.0001
	Negative	0	612 (94.15%)		

^†^The calculation method for diagnostic yield results of RT-qPCR as follows: (positive detections by RT-qPCR only) / (positive detections by RT-qPCR and culture + positive detections by culture only).

### 3.4 Co-infection

**FIGURE 2:**
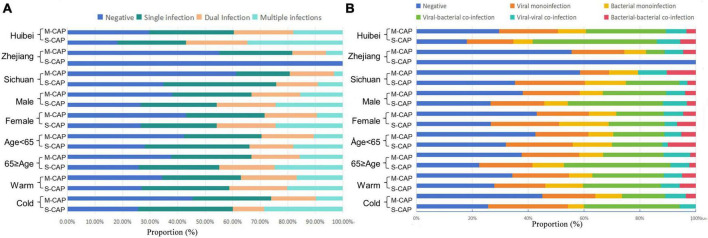
The proportions of different types of co-infections among patients with M-CAP and S-CAP. **(A)** Positive proportion of Negative, Single infection, Dual Infection and Multiple infections in different groups. **(B)** Positive proportion of Negative, viruses, bacteria, viral–bacteria co-infections, viral–viral co-infections and bacterial–bacterial co-infections in different group.

In this study, dual infection is defined as the simultaneous presence of two different pathogens in a single patient, while multiple infection is defined as the simultaneous presence of more than two different pathogens in a single patient. Among the 650 patients, 146 (22.46%) had monoinfection, while 158 (24.31%) had co-infections. Among these co-infections, 15.38% (100) were viral-bacterial co-infections, 3.23% (21) were bacterial-bacterial co-infections, and 5.69% (37) were viral-viral co-infections. Additionally, 14.15% (92/650) of the co-infections were classified as dual infections, and 10.15% (66/650) were classified as multiple infections. Overall, the rates of multiple infections (22.30% [31/139] vs 12.92% [66/511]) and viral-bacterial co-infections (30.22% [42/139] vs 19.57% [100/511]) were higher in S-CAP compared to M-CAP. Specifically, in the Hubei region (34.72% [25/72] vs 18.00% [54/300]), among males (24.47% [23/94] vs 15.47% [41/265]), and during the Cold season (28.57% [10/35] vs 9.86% [28/284]), the rate of multiple infections was higher in S-CAP than in M-CAP. Similarly, in the Hubei region (43.06% [31/72] vs 29.00% [87/300]), Sichuan region (16.67% [11/66] vs 0% [0/31]), and among males (35.11% [33/94] vs 22.64% [60/265]), the rate of viral-bacterial co-infections was higher in S-CAP than in M-CAP. However, no difference in the rate of viral-viral co-infections was observed between S-CAP and M-CAP in any group. Additionally, no difference in the types of co-infections during the warm season was observed between S-CAP and M-CAP (Figure 2 and Supplementary Table 3).

**FIGURE 3:**
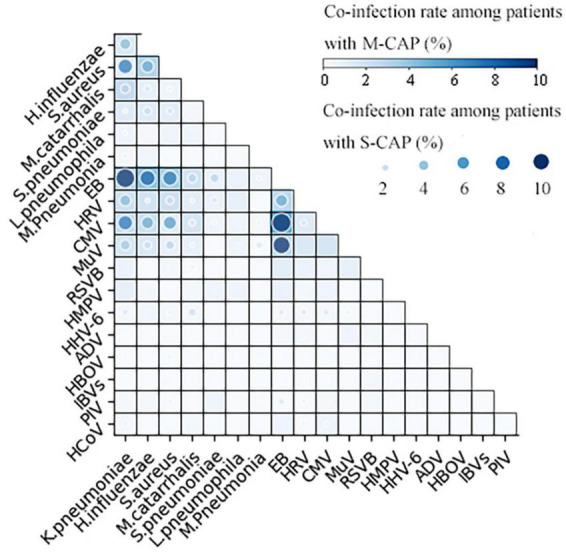
Heatmap of the co-infection rates of respiratory pathogens. The grid color represents the co-infection rate of respiratory pathogens among patients with M-SCAP, and the dot color represents the co-infection rate of respiratory pathogens among patients with S-CAP. Larger and darker dots indicate higher co-infection rates between the respective pairs of pathogens.

Among the co-infections, the most frequent bacterial ones were *S. aureus-K. pneumoniae*, *M. catarrhalis-K. pneumoniae*, and *S. aureus*-*H. influenzae*. As for viral co-infections, the most common were EB-HRV, EB-CMV, and EB-MuV. Bacterial and viral co-infections frequently encountered comprised EB- *K. pneumoniae*, EB- H. influenzae, and EB- *S. aureus*. With the exception of *M. catarrhalis*-*K. pneumoniae* co-infections in S-CAP cases (2.88% [4/139] vs. 2.35% [12/511]), which were slightly higher than in M-CAP cases, all others were significantly higher (*p* < 0.05) (Figure 3).

### 3.5 Estimating the impact of the RT-qPCR assays on the prescription of antimicrobials

Hospital-administered antimicrobial treatment records were available for 518 (79.69%) patients. According to the test results, 22.59% (117/518) of the patients received sub-optimal initial antibiotic treatment. Potentially, the RT-qPCR could permit de-escalation in the spectrum and/or number of the initial empirical antibiotic agents in 18.30% (95/518) of patients, an increase in the spectrum and/or number in 2.51% (13/518) of patients (Table 4). Swapping the class of antibiotics chiefly meant swapping 2nd or 3rd generation cephalosporins, quinolones, or combinations of β-lactamase inhibitors for macrolides. Reducing the number of antibiotics chiefly comprised decreasing the use of 2nd or 3rd generation cephalosporins (5.79% [30/518]) and β-lactamase inhibitor combinations (2.51%, [13/518]).

**TABLE 4:** Guiding antibiotic treatment using the results of RT-qPCR assays (*n* = 518).

Potential Modification	Anti-infective agents for initial empirical therapy	*N* (%)
De-escalation		95 (18.30%)
Remove 1 agent		55 (10.62%)
	β-lactamase inhibitor combinations	13 (2.51%)
	2nd or 3rd generation cephalosporins	30 (5.79%)
	Quinolones	10 (1.93%)
	Tetracyclines	2 (0.39%)
Remove 2 agents		40 (7.72%)
	β-lactamase inhibitor combinations + II or III generation cephalosporins	3 (0.58%)
	β-lactamase inhibitor combinations + Quinolones	5 (0.97%)
	2nd or 3rd generation cephalosporins + Quinolones	32 (6.18%)
Escalation		13 (2.51%)
Add 1 agent		13 (2.51%)
	2nd or 3rd generation cephalosporins	1 (0.19%)
	Macrolides	7 (1.35%)
	Quinolones	3 (0.58%)
	Tetracyclines	2 (0.39%)
Reduce number and spectrum		9 (1.74%)
	2nd or 3rd generation cephalosporins + Quinolones to macrolides	5 (0.97%)
	β-lactamase inhibitor combinations + 2nd or 3rd generation cephalosporins to macrolides	2 (0.39%)
	β-lactamase inhibitor combinations to macrolides	2 (0.39%)
No change		401 (77.41%)

## 4 Discussion

In this investigation of patients with CAP, we delineated the epidemiological characteristics of CAP, identified the rates of pathogen infection in patients with S-CAP and M-CAP, and examined the patterns of co-infection. Additionally, we explored variations by region, gender, age, and season. The observed higher incidence of S-CAP in males, older adults, and during the warm season highlights the necessity for targeted preventive strategies within these populations. Furthermore, the variation in pathogen prevalence across different regions and seasons indicates that local epidemiological data should inform the empirical treatment of CAP.

*K. pneumoniae*, *H. influenzae*, and *S. aureus* are considered to be the primary bacterial pathogens, while the detection of viral pathogens such as EB, HRV, and CMV should not be overlooked (Templeton et al., 2004; Cantan et al., 2019). These findings resonate with trends observed in previous research, suggesting their potential significance in the etiology of CAP. The higher rates of virus infection among males and older individuals may be closely associated with their physiological and immune system characteristics (Channappanavar and Perlman, 2020; Liu et al., 2023). As individuals age, their immune responses may weaken, which also explains why older individuals are more susceptible to virus infections. Furthermore, factors such as lifestyle and behavioral habits may contribute to explaining these epidemiological patterns. In general, males are more susceptible to the severe consequences of respiratory virus infections (Ygberg et al., 2022).

Understanding mixed respiratory infections is a crucial and continuously evolving field in epidemiological and clinical research (Jiang et al., 2017). In our investigation, a substantial number of patients demonstrated co-infections, primarily attributed to the detrimental effects of viral infections on the airways, promotion of bacterial adhesion, and disruption of the host’s immune system equilibrium, thereby fostering bacterial proliferation. Conversely, bacterial infections alter viral transmission and invasion mechanisms, heightening the host’s vulnerability to viral infections (Choi et al., 2012; Borchers et al., 2013; Karhu et al., 2014; Voiriot et al., 2016; Lim et al., 2019). Within cases of S-CAP, a notable prevalence of polymicrobial infections and virus-bacteria co-infections was identified, suggesting that co-infections may exacerbate disease severity. Noteworthy patterns of co-infections were observed, including frequent combinations of EB virus with *K. pneumoniae* and *S. aureus* bacterial pathogens. These discoveries emphasize the intricate nature of CAP etiology and stress the significance of comprehensive diagnostic strategies to precisely identify co-infecting pathogens, which could substantially impact treatment decisions and prognosis.

This study demonstrated that the use of RT-qPCR almost doubled the pathogen detection rate in patients with CAP. In particular, *K. pneumoniae* and *H. influenzae* ranked as the third and fourth most common pathogens, respectively, with identification rates over 10%. This detection rate was higher than that in studies in which bacteria were detected using culture. Therefore, culture-based testing would underestimate the spectrum of the underlying causative pathogens, which would result in misapprehension of the risk of bacterial infection and missed diagnoses (Zhang et al., 2015). RT-qPCR, can report results within 1 day. Therefore, they permit the guided selection of antimicrobials (Templeton et al., 2004; Vijgen et al., 2005; Sterback et al., 2013). Reviews have stated that reducing the excessive prescription of antibiotics and increasing the use of effective antibiotics in patients with CAP positively affects antimicrobial resistance (Davey et al., 2013; Gadsby et al., 2016; Xiao et al., 2016). In this study, we found that the number and/or spectrum of initial empirical antibiotic agents was enhanced in clinical treatment, which would increase the patients’ economic burden and the antimicrobial resistance of respiratory pathogens (Gandra et al., 2014). Therefore, leveraging high-precision and rapid detection techniques can better guide the use of antibiotics in CAP patients, thus more effectively managing CAP and reducing its public health implications.

Our study had several limitations. First, detailed information, e.g., lifestyle, socioeconomic factors, and previous hospitalization history, was not available. Second, despite trying to identify as many CAP-causing pathogens as possible, the RT-qPCR has a limited ability to discover all causative pathogens. Third, many factors affect the selection of antimicrobials, e.g., illness severity, drug allergy or inflammatory considerations. However, the results of our study provide physicians with a marked increase in information that could be used to make treatment decisions.

## 5 Conclusion

We believe that the RT-qPCR can identify pathogens in CAP more comprehensively and earlier, which will lead to improved culture conditions and antibiotic regimens.

## Data availability statement

The original contributions presented in this study are included in the article/Supplementary material, further inquiries can be directed to the corresponding author.

## Ethics statement

The studies involving humans were approved by the Ethical Committee of Communicable Disease Control and Prevention, Chinese Center for Disease Control and Prevention (CDC), the China (No. ICDC-2019012). The studies were conducted in accordance with the local legislation and institutional requirements. The participants provided their written informed consent to participate in this study. Written informed consent was obtained from the individual(s) for the publication of any potentially identifiable images or data included in this article.

## Author contributions

NZ: Writing – original draft, Writing – review and editing. HR: Writing – original draft. YZ: Resources, Writing – review and editing. YJ: Writing – original draft. JD: Resources, Writing – review and editing. LJ: Resources, Writing – review and editing. ZL: Resources, Writing – review and editing. TQ: Conceptualization, Writing – review and editing.
