# Supplementary material for: Enhancing pathogen description and antibiotic regimen selection in community-acquired pneumonia through RT-qPCR assays
Source: Front Microbiol. 2024 Jun 11;15:1409065. doi: 10.3389/fmicb.2024.1409065 (PMC11196416; doi:10.3389/fmicb.2024.1409065)
Supplement: Supplementary file 1 [file Data_Sheet_1.doc]

Supplementary Material

Supplementary [Table 1](https://www.ncbi.nlm.nih.gov/pmc/articles/PMC8335481/figure/f2/). Respiratory tract microbiota targets

| Pathogen type | Target pathogen | Pathogen type | Target pathogen | Pathogen type | Target pathogen | Pathogen type | Target pathogen |
| --- | --- | --- | --- | --- | --- | --- | --- |
| Bacteria | *Bordetella* | Bacteria | *Mycoplasma pneumoniae* | Virus | Influenza A (Pan) | Virus | Bochavirus |
|  | *Bordetella holmesii* | Fungus | *Pneumocystis jirovecii* |  | Influenza A/H1 |  | Epstein-Barr virus |
|  | *Bordetella pertussis* | Virus | Adenovirus |  | Influenza A/H3 |  | SARS-CoV |
|  | *Chlamydia pneumoniae* |  | Coronavirus HKU1 |  | Influenza B (Pan) |  | MERS-CoV |
|  | *Haemophilus influenzae* |  | Coronavirus NL63 |  | Parainfluenza 1 |  | Mumps |
|  | *Klebsiella pneumoniae* |  | Coronavirus 229E |  | Parainfluenza 2 |  | Measles |
|  | *Coxiella burnetii* |  | Coronavirus OC43 |  | Parainfluenza 3 |  | Cytomegalovirus |
|  | *Staphylococcus aureus* |  | Rhinovirus |  | Parainfluenza 4 |  | Human herpesvirus 6 |
|  | *Streptococcus pneumoniae* |  | Human metapneumovirus |  | Respiratory Syncytial Virus A |  | Varicella zoster virus |
|  | *Legionella pneumophila* |  | Enterovirus |  | Respiratory Syncytial Virus B | Control | Bacillus atrophaeus |
|  | *Moraxella catarrhalis* |  | Enterovirus D68 |  | Parechovirus |  | RNase P |
|  |  |  |  |  |  |  | 18s |
|  |  |  |  |  |  |  | Xeno |

[Table S2](https://www.ncbi.nlm.nih.gov/pmc/articles/PMC8335481/figure/f2/). Selection of anti-infective agents for initial empirical therapy for patients treated as inpatients

| Populations | Common pathogens | Anti‐infective agents for initial empirical therapy |
| --- | --- | --- |
| Young adults without underlying disease(s) | *S. pneumoniae, H. influenzae*, *M. catarrhalis*, *S. aureus*, *M. pneumoniae*, *C. pneumoniae*, *influenza virus*, *adenovirus*, other *respiratory tract viruses* | (1) Penicillin G, aminopenicillins, penicillins‐β‐lactamase‐inhibitor combinations;  (2) II or III generation cephalosporins, cephamycins, oxacephems;  (3) the above drugs combined with doxycycline, minocycline or macrolides;  (4) respiratory quinolones;  (5) macrolides |
| Patients with underlying disease(s) or elderly patients (age ≥ 65 years) | *S. pneumoniae*, *H. influenzae*, *Enterobacteriaceae* such as *K. pneumoniae*, *influenza virus*, *RSV*, M. catarrhalis, anaerobic bacteria, Legionella | (1) Penicillins‐β‐lactamase‐inhibitor combinations;  (2) III generation cephalosporins or their enzyme‑inhibitor combinations, carbapenems such as cephamycins, oxacephems, ertapenem;  (3) monotherapy with the above drugs or in combination with macrolides;  (4) respiratory quinolones |

II generation cephalosporins: e.g., cefuroxime, cefamandole, cefotiam, cefaclor, and cefprozil. III generation cephalosporins: intravenous: e.g., ceftriaxone, cefotaxime, and ceftizoxime; oral: e.g., cefdinir, cefixime, cefpodoxime proxetil, and cefditoren pivoxil; Macrolides: azithromycin, clarithromycin, and erythromycin; Respiratory quinolones: levofloxacin, moxifloxacin, and gemifloxacin. Aminopenicillins: amoxicillin, and ampicillin; Penicillins‐β‐lactamase‐inhibitor combinations (not including penicillins with antipseudomonal activity, such as piperacillin, ticarcillin): e.g., amoxicillin‐clavulanic acid, amoxicillin‐sulbactam, and ampicillin‐sulbactam; Cephamycins: cefoxitin, cefmetazole, cefotetan, and cefminox.

Supplementary table 3. Co-infection rate compared between M-CAP and S-CAP patients

|  | Area group | | | | | | | | | Sex group | | | | | |
| --- | --- | --- | --- | --- | --- | --- | --- | --- | --- | --- | --- | --- | --- | --- | --- |
|  | HuBei (n=372) | | | Zhejiang (N =181) | | | Sichuan(N =97 ) | | | Male(N =359) | | | Female(N =291) | | |
|  | M-CAP (n=300) | S-CAP（n=72） | P value | M-CAP （n=180） | S-CAP（n=1） | P value | M-CAP(n=31) | S-CAP(n=66) | P value | M-CAP (n=265) | S-CAP(n=94) | P value | M-CAP(n=246) | S-CAP(n=45) | P value |
| Negative | 29.67% | 18.06% | 0.047 | 55.00% | 100.00% | 1 | 61.29% | 34.85% | 0.014 | 38.11% | 26.60% | 0.039 | 43.09% | 26.67% | 0.044 |
| Single infection | 30.67% | 25.00% | 0.344 | 26.67% | 0.00% | 1 | 19.35% | 40.91% | 0.037 | 28.68% | 27.66% | 0.851 | 28.46% | 42.22% | 0.065 |
| Dual Infection | 21.67% | 22.22% | 0.918 | 12.22% | 0.00% | 1 | 16.13% | 15.15% | 1 | 17.74% | 21.28% | 0.449 | 19.11% | 13.33% | 0.356 |
| Multiple infections | 18.00% | 34.72% | 0.002 | 6.11% | 0.00% | 1 | 3.23% | 9.09% | 0.535 | 15.47% | 24.47% | 0.05 | 9.35% | 17.78% | 0.155 |
| Viral monoinfection | 21.00% | 16.67% | 0.41 | 18.89% | 0.00% | 1 | 9.68% | 25.76% | 0.068 | 20.38% | 19.15% | 0.798 | 18.70% | 24.44% | 0.372 |
| Bacterial monoinfection | 9.67% | 8.33% | 0.728 | 7.78% | 0.00% | 1 | 9.68% | 15.15% | 0.676 | 8.30% | 8.51% | 0.95 | 9.76% | 17.78% | 0.186 |
| Viral-bacterial co-infection | 29.00% | 43.06% | 0.021 | 7.22% | 0.00% | 1 | 0.00% | 16.67% | 0.038 | 22.64% | 35.11% | 0.018 | 16.26% | 20.00% | 0.538 |
| Viral-viral co-infection | 7.33% | 8.33% | 0.773 | 6.67% | 0.00% | 1 | 9.68% | 4.55% | 0.599 | 6.79% | 7.45% | 0.83 | 7.72% | 4.44% | 0.64 |
| Bacterial-bacterial co-infection | 3.33% | 5.56% | 0.586 | 4.44% | 0.00% | 1 | 9.68% | 3.03% | 0.374 | 3.77% | 3.19% | 1 | 4.47% | 6.67% | 0.8 |

Supplementary table 3 (Continued)

|  | Age group | | | | | | Season of infection | | | | | | Total (N =650) | | |
| --- | --- | --- | --- | --- | --- | --- | --- | --- | --- | --- | --- | --- | --- | --- | --- |
|  | Age < 65(N =362) | | | 65 ≤ age (N =288) | | | Warm cases (N =331) | | | Cold cases (N =319) | | |  |  |  |
|  | M-CAP（n=312） | S-CAP(n=199) | P value | M-CAP(n=199) | S-CAP(n=89) | P value | M-CAP(n=227) | S-CAP(n=104) | P value | M-CAP(n=284) | S-CAP(n=35) | P value | M-CAP(n=511) | S-CAP(n=139) | P value |
| Negative | 42.31% | 28.00% | 0.003 | 37.69% | 25.84% | 0.05 | 34.36% | 26.92% | 0.178 | 45.42% | 25.71% | 0.026 | 40.51% | 26.62% | 0.003 |
| Single infection | 28.21% | 38.00% | 0.038 | 29.15% | 29.21% | 0.991 | 28.63% | 31.73% | 0.567 | 28.52% | 34.29% | 0.479 | 28.57% | 32.37% | 0.383 |
| Dual Infection | 18.91% | 16.00% | 0.957 | 17.59% | 20.22% | 0.594 | 20.26% | 21.15% | 0.852 | 16.20% | 11.43% | 0.464 | 18.00% | 18.71% | 0.849 |
| Multiple infections | 10.58% | 18.00% | 0.052 | 15.58% | 24.72% | 0.064 | 16.74% | 20.19% | 0.446 | 9.86% | 28.57% | 0.003 | 12.92% | 22.30% | 0.006 |
| Viral monoinfection | 19.23% | 24.00% | 0.195 | 20.10% | 19.10% | 0.844 | 20.26% | 18.27% | 0.671 | 19.01% | 28.57% | 0.183 | 19.57% | 20.86% | 0.735 |
| Bacterial monoinfection | 8.97% | 14.00% | 0.223 | 9.05% | 10.11% | 0.774 | 8.37% | 13.46% | 0.151 | 9.51% | 5.71% | 0.671 | 9.00% | 11.51% | 0.372 |
| Viral-bacterial co-infection | 18.27% | 18.00% | 0.684 | 21.61% | 37.08% | 0.006 | 25.55% | 28.85% | 0.529 | 14.79% | 34.29% | 0.004 | 19.57% | 30.22% | 0.007 |
| Viral-viral co-infection | 6.09% | 6.00% | 1 | 9.05% | 6.74% | 0.513 | 6.61% | 6.73% | 0.967 | 7.75% | 5.71% | 0.928 | 7.24% | 6.47% | 0.755 |
| Bacterial-bacterial co-infection | 5.13% | 10.00% | 0.181 | 2.51% | 1.12% | 0.752 | 4.85% | 5.77% | 0.734 | 3.52% | 0.00% | 0.026 | 4.11% | 4.32% | 0.914 |
